# Transarterial Chemoembolization Combined With Immune Checkpoint Inhibitors and Tyrosine Kinase Inhibitors for Unresectable Hepatocellular Carcinoma: Efficacy and Systemic Immune Response

**DOI:** 10.3389/fimmu.2022.847601

**Published:** 2022-02-18

**Authors:** Fei Yang, Gui-Li Xu, Jin-Tao Huang, Yu Yin, Wei Xiang, Bin-Yan Zhong, Wan-Ci Li, Jian Shen, Shuai Zhang, Jun Yang, Hong Peng Sun, Wan-Sheng Wang, Xiao-Li Zhu

**Affiliations:** ^1^ Department of Interventional Radiology, The First Affiliated Hospital of Soochow University, Suzhou, China; ^2^ Department of Interventional Radiology, Affiliated Hospital of Jiangnan University, Wuxi, China; ^3^ Department of Oncology Intervention, Taizhou Municipal Hospital, Taizhou, China; ^4^ Jiangsu Key Laboratory of Preventive and Translational Medicine for Geriatric Diseases, School of Public Health, Soochow University, Suzhou, China

**Keywords:** hepatocellular carcinoma, TACE, immune checkpoint inhibitors, tyrosine kinase inhibitors, humoral immune response, B cells

## Abstract

**Background:**

Locoregional therapy combined with systemic therapy can further improve the prognoses for HCC. However, the efficacy of TACE combined with ICIs and TKIs for HCC and whether this triple therapy can activate systemic immune response are still unknown.

**Purpose:**

To identify the efficacy of TACE+ICIs+TKIs for unresectable hepatocellular carcinoma (uHCC) and its effect on systemic immunity.

**Materials and Methods:**

This single-center retrospective study was approved by the Institutional Review Board. From August 1, 2019, to March 30, 2021, patients with uHCC who received the combination therapy of TACE+ICIs+TKIs were included. Peripheral blood samples were collected at baseline and once a month for 4 months after treatment. Lymphocyte subsets were measured by flow cytometry. Immunoglobulins were measured using the immune turbidimetric method. The dynamic change trend of circulating parameters was tested using simple linear regression.

**Results:**

Fifty-three patients with a mean age of 59 ± 10.6 years were included. TTP was 8.0 months (95% CI, 5.5–10.5) and PFS was 8.5 months (95% CI, 5.4–11.5). ORR was 52.8% and DCR was 81.1%. Twenty patients had completed analysis of biomarkers in peripheral blood. For cellular immune response, the level of circulating CD8^+^, CD3^+^ T cells and NK cells increased, the frequency of CD4^+^T cells and the CD4^+^/CD8^+^ ratio decreased, and among them, CD8^+^ T cells increased significantly. For humoral immune response, there was a significant decrease in B cells and a significant increase in Ig G, Ig κ, and Ig λ. Moreover, Ig G, Ig κ, and Ig λ were related to tumor response.

**Conclusion:**

TACE+ICIs+TKIs showed considerable efficacy in patients with uHCC. This triple therapy activated not only cell immune but also humoral immune activation. Circulating Ig G, Ig λ, and Ig κ can serve as potential biomarkers.

## Introduction

The prognosis of hepatocellular carcinoma (HCC) still needs to be improved. Only approximately 30% of HCC patients are diagnosed early enough to benefit from surgical resection (1). For patients with unresected HCC (uHCC), transarterial therapies are the most commonly used treatment and can be divided into TAE (bland embolization without chemotherapy drugs), TACE (lipiodol mixed with chemotherapy drugs), dTACE (drug-eluting bead), and TARE (radioactive particles containing yttrium-90), according to different embolization materials. Although there is no clear evidence to prove which embolization method is more effective (2), major guidelines recommend TACE as the standard method of treatment (3–5). However, the complete response (CR) rate of TACE is limited (6), and residual tumor often require repeated TACE. The response rate of TACE diminishes as the number of TACE sessions increases, and if three sessions of TACE are still ineffective, treatment should be halted (7). Furthermore, for patients with a high tumor burden, a combination of systemic therapy is frequently required (8).

Tyrosine kinase inhibitors (TKIs) and immune checkpoint inhibitors (ICIs) are two main systemic therapies for HCC, with a monotherapy response rate of less than 20% (9). The combination of ICIs with TKIs has revealed surprisingly positive results (10, 11). The major mechanism of ICIs plus TKIs is to modify the hypoxia and immunosuppressive tumor microenvironment (TME) by normalizing the tumor blood vessels (12). “Vascular normalization”has the potential to improve therapeutic agents’ delivery and, more importantly, to reverse immunosuppressive TME by promoting effected T-cell infiltration into TME, maturation of antigen-presenting cells (such as DCs), and reduction of immunosuppressive factors such as MDSCs and VEGF (12). Therefore, TKIs enhance the efficacy of ICIs *via* vascular normalization. Remarkably, ICI treatment may promote tumor vascular normalization. ICIs activate IFN*γ^+^* Type 1 T helper (Th1) cells, which are the major population related with tumor vascular normalization (13). TACE, by transarterial embolization, can worsen the hypoxic milieu of remnant viable tumors. Subsequent angiogenesis occurs by proangiogenic cytokines, such as VEGF. Hypoxia and overexpression of VEGF lead to an immunosuppressive TME (14). On one hand, TACE aggravates hypoxia and immunosuppressive TME. On the other hand, ICIs plus TKIs can modify the hypoxia and immunosuppressive TME. As a result, TACE combined with TKIs and ICIs may have the potential to further improve the efficacy of uHCC.

In addition to local tumor destruction, locoregional therapy has been proven to have a systemic immune response. For example, PD-1 expression in peripheral mononuclear cells increased and the ratio of CD4+/CD8+ cells was reduced after TACE (15). Tremelimumab (anti-CTLA-4) was combined with RFA or TACE for HCC, and peripheral blood CD8^+^ T cells were observed to increase 2-fold over baseline and lasted for at least 12 weeks (16). Tregs were significantly lower after TACE than that before TACE (17). These data are based on the cellular side of the systemic immune response. In addition, recent groundbreaking studies have shown that B cells are associated with the response to ICI immunotherapy in cancer patients (18, 19). B cells constitute the humoral arm of systemic immunity. Therefore, we hypothesized that the combination of TACE+ICIs+TKIs may have significant effects on both cellular and humoral immune responses.

In this study, we tried to confirm the efficacy of TACE+TKIs+ICIs for uHCC and to explore both cellular and humoral immune responses induced by this triple combination therapy.

## Materials and Methods

### Patients

This single-center retrospective study was approved by the Institutional Review Board of the First Affiliated Hospital of Soochow University (Suzhou, Jiangsu Province, China). Informed written consent was obtained from all patients. Patients were diagnosed according to the European Society for Medical Oncology (ESMO) guideline (3). Triple combination therapy of TACE+ICIs+TKIs was defined as patients administered ICIs and TKIs within 1 month before or after TACE (20, 21). Other inclusion criteria included the following: (1) 18–80 years old, (2) Eastern Cooperative Oncology Group performance status (ECOG-PS) 0–1, (3) Child–Pugh class A or B, and (4) with at least one measurable target lesion on baseline CT or MRI. Exclusion criteria were as follows: (1) with other malignant tumors, (2) targeted lesion received other locoregional therapy, such as ablation and brachytherapy, or (3) without at least one set of follow-up image data.

### Treatments

TACE was performed according to standard procedures (5). All TACE procedures were performed by 3 different interventional radiologists, each with 25 years, 10 years, and 8 years of experience (X-LZ, W-SW, and JS). The right femoral artery was punctured using the Seldinger method, and angiography of the celiac artery and superior mesenteric artery was performed to confirm the collateral blood supply. Intraprocedural cone-beam CT (CBCT) was used to identify tumor-feeding arteries. A 2.3 Fr microcatheter was used to catheterize these tumor-feeding arteries. Embolization was performed as selectively as possible. Following CBCT confirmation, an emulsion of lipiodol and chemotherapeutic agent (THP 10–20 mg) was injected into tumor-feeding arteries. Lipiodol consumption was less than 30 ml. If necessary, additional embolization with gelatin sponge or polyvinyl alcohol particles (300–500 μm) was conducted until there was no tumor staining. Repeated TACE was performed in the on-demand manner. ICI immunotherapy used camrelizumab (200 mg) every 3 weeks intravenously. TKI therapy used sorafenib (800 mg)/lenvatinib (8 mg or 12 mg) orally daily. Fixed-dose administration of camrelizumab was used until disease progression or unexpected toxicity. The dose and interval of TKIs allowed changes depending on toxicity and disease conditions. Systematic therapy was suspended during the TACE procedure and resumed 3–7 days after TACE.

### Follow-up

Multiphase enhanced CT or MRI was performed before treatment, 1–3 months after initial treatment, and every 2–3 months thereafter. Tumor response was evaluated by the investigator (X-LZ, with 25 years of experience) according to the Modified Response Evaluation Criteria in Solid Tumors (mRECIST) (22). The endpoints of the study included objective response rate (ORR), disease control rate (DCR), time to progression (TTP), and progression-free survival (PFS). TTP was calculated from the date of first TACE to radiological progression. If patients died without radiological progression, data were censored at the date of final imaging evaluation. PFS was calculated from the date of first TACE to radiological progression or death. If patients were still alive without radiological progression, data were censored at the cutoff day.

Treatment-related adverse events (AEs) were graded according to the National Cancer Institute Common Toxicity Criteria Adverse Events (CTCAE) version 5.0. To avoid interference from postembolization syndrome, AEs were assessed at least 1 month after TACE.

### Explore Systemic Immune Response in Peripheral Blood

Peripheral blood samples were collected before TACE and monthly during treatment (before ICI therapy or TACE procedure). Peripheral blood mononuclear cells (PBMCs) were isolated using the Ficoll-Paque method. Cells were resuspended at 1 × 10^6^/ml, and the PBMCs were harvested and measured by flow cytometry (FACScan Caliber, Becton Dickinson, Franklin Lakes, NJ, USA). The antibodies were as follows: FITC-conjugated anti-CD3 antibody, PE-Cy™7-conjugated anti-CD4 antibody, APC-Cy7-conjugated anti-CD8 antibody, PE-conjugated CD16 antibody, PE-conjugated CD56 antibody, and PAPC-conjugated anti-CD19 antibody. Immunoglobulins and complements were measured using the immune turbidimetric method. Serum samples were collected at the same time as PBMCs. Antibodies (coating antibody, Beckman Coulter, Inc, USA) were as follows: Ig G, Ig M, Ig A, Ig κ, Ig λ, complement C3, complement C4, and *B* factor. The protocol was performed according to the manufacturer’s instructions.

### Statistical Analysis

Continuous variables are expressed as means with SDs. For categorical variables, counts and percentages are presented. TTP and PFS were estimated using Kaplan–Meier analysis. The dynamic change trend of circulating parameters was tested using simple linear regression. For the difference among baseline, ORR, and PD, Shapiro–Wilk tests were performed to determine the normality of the data distribution. When data were normally distributed, one-way analysis of variance (ANOVA) or unpaired *t*-test was used to determine the significant differences. When data from any cohort were not normally distributed, Kruskal–Wallis one-way analysis or the Mann-Whitney test was used.

All analyses were conducted with SPSS version 26 software (IBM Corp., Armonk, NY, USA). The level of significance was set at a 2-sided *p*-value <0.05. The photograph was drawn using GraphPad Prism 8 (GraphPad Software, CA).

## Results

### Patient Characteristics

**Figure 1:**
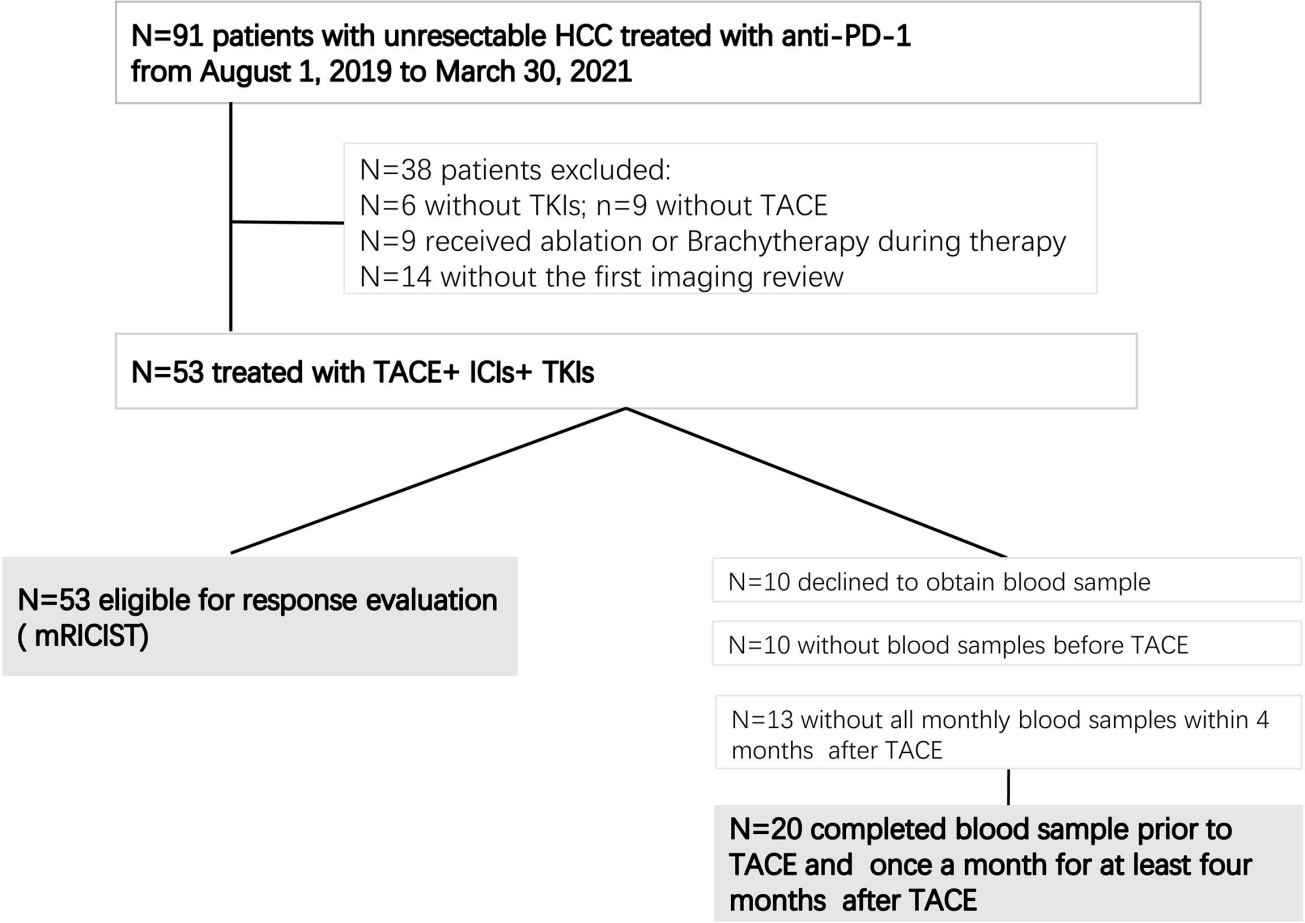
Patient flow chart.

From August 1, 2019, to March 30, 2021, a total of 53 patients with uHCC met the inclusion criteria and were included, all of whom received combination therapy with TACE+ICIs+TKIs (**Figure 1**). Among them, 20 patients had blood samples collected at baseline and once a month for 4 months after initiation of the treatment. Patient characteristics are summarized in **Table 1**.

**Table 1:** Baseline patient demographics.

Characteristics	All patient (*n* = 53)	Cohorts for biomarker evaluation (*n* = 20)
Age (years), means ± SD	59 ± 10.6	59 ± 11.2
Sex		
Female	8 (15)	3 (15)
Male	45 (85)	17 (85)
Etiology		
Hepatitis B	47 (89)	19 (95)
Others	6 (11)	1 (5)
Child-Pugh class		
A	47 (89)	19 (95)
B	6 (11)	1 (5)
AFP		
<400 (IU/ml)	34 (64)	11 (55)
≥400 (IU/ml)	19 (36)	9 (45)
Tumor distribution		
One hepatic lobe	36 (68)	16 (80)
Two hepatic lobes	17 (32)	4 (20)
Tumor burden		
<6	11 (20)	4 (20)
≥6, <12	20 (38)	5 (25)
≥12	22 (42)	11 (55)
BCLC		
A	2 (4)	1 (5)
B	29 (54)	9 (45)
C	22 (42)	10 (50)
ECOG PS		
0	29 (55)	12 (60)
1	24 (45)	8 (40)
Immunotherapy		
First-line	35 (67)	16 (80)
Second-line	14 (26)	3 (15)
Third-line	4 (7)	1 (5)

BCLC, Barcelona Clinic Liver Cancer (BCLC) stage; ECOG PS, Eastern Cooperative Oncology Group performance status score. Data are numbers with percentages, or presented as means ± SD.

### Efficacy

The median number of TACE procedures per patient was 2 (range, 1–7). The median time of ICI immunotherapy was 5 cycles (range, 1–20 cycles). The median time interval between TACE and ICIs was 14.32 days (range, 3–28). Twenty patients received TKIs before TACE, and the other 33 patients received TKIs after TACE. For the latter, the interval between TACE and TKIs was 5.7 days (range, 5–11). Thirty-four patients received sorafenib, and 19 patients received lenvatinib.

At the cutoff date of July 31, 2021, 18 patients had died with a median follow-up period of 10.8 months (range, 1.5–24.2). TTP was 8.0 months [95% confidence interval (CI), 5.5–10.5], and PFS was 8.5 months (95% CI, 5.4–11.5). As the best treatment response, 10 patients had a complete response (18.9%) and 18 patients achieved a partial response (33.9%). Thus, the objective response rate (ORR) was 52.8%. Fifteen patients had stable disease (28.3%), resulting in a disease control rate (DCR) of 81.1% (**Table 2**).

**Table 2:** Radiological response according to mRECIST and clinical efficacy.

Best response	Number	Percentage (%)
CR	10	18.9
PR	18	33.9
SD	15	28.3
PD	10	20.7
ORR (CR+PR)	28	52.8
DCR (CR+PR+SD)	43	81.1

TTP, median (95% CI) 8.0 (5.5–10.5) months.

PFS, median (95% CI) 8.5 (5.4–11.5) months.

### Safety

Forty-five (85%) patients experienced at least one AE during the treatment (**Table 3**). Incidence greater than 20% includes reactive capillary endothelial proliferation (RCCEP; *n* = 18; 34%), hypothyroidism (*n* = 11; 21%), total bilirubin increase (*n* = 21; 40%), alanine aminotransferase increase (*n* = 12; 23%), aspartate aminotransferase increase (*n* = 12; 23%), and proteinuria (*n* = 11; 21%). Fifteen (28%) patients developed AEs of higher grade(grade ≥3). Three patients discontinued camrelizumab due to grade 3 gastrointestinal bleeding, myocarditis, and hypophysitis. No patient died due to AEs.

**Table 3:** Adverse events.

Event	Any grade *n* (%)	Grade 3 or 4 *n* (%)
Rash	10 (18)	–
Pruritus	6 (11)	–
Reactive capillary endothelial proliferation	18 (34)	–
Hoarse voice	2 (3)	1 (2)
Nausea	4 (7)	1 (2)
Diarrhea	8 (15)	–
Hand–foot syndrome	8 (15)	–
Pyrexia	4 (7)	1 (2)
Hypertension	9 (17)	1 (2)
BNP	4 (7)	2 (3)
Hypothyroidism	11 (21)	–
Blood Total bilirubin increase	21 (40)	2 (3)
Alanine aminotransferase increase	12 (23)	1 (2)
Aspartate aminotransferase increase	12 (23)	–
Albumin decrease	6 (11)	–
Proteinuria	11 (21)	1 (2)
Cardiac troponin	6 (11)	–
Myocarditis	–	1 (2)
Hypophysitis	2 (3)	1 (2)
Platelet count decrease	2 (3)	1 (2)
Drowsy	1 (2)	1 (2)
Gastrointestinal bleeding	3 (5)	1 (2)
Allergy	2 (3)	1 (2)

### Systemic Immune Response in Peripheral Blood

At baseline, cellular immune components in peripheral blood were similar to other reports (23). The percentages of total T cells (CD3^+^), CD4^+^T cells (CD3^+^CD4^+^), CD8^+^T cells (CD3^+^CD8^+^), and NK cells (CD3^-^CD16^+^CD56^+^) were 72.57%, 46.12%, 22.85%, and 15.71%, respectively. For humoral immunity components, the percentage of total B cells (CD3^-^CD19^+^) was 10.79%. The absolute numbers of kappa light chains (Ig κ), lambda light chains (Ig λ), Ig A, Ig G, Ig M, complement C3, C4, and *B* factor are listed in **Table 4**.

**Table 4:** Percentage and number of biomarkers in peripheral blood.

	0 m	1 m	2m	3 m	4m	*p*-value
	Mean (SD)	Mean (SD)	Mean (SD)	Mean (SD)	Mean (SD)	
CD3^+^(%)	72.57 (9.83)	76.98 (8.01)	74.17 (10.09)	74.32 (10.73)	77.98 (4.19)	0.23
CD4^+^/CD8^+^(%)	2.14 (0.92)	2.13 (0.84)	2.11 (0.89)	1.92 (0.75)	1.83 (0.87)	0.13
CD8^+^(%)	22.53 (6.04)	24.81 (6.91)	23.84 (6.80)	25.41 (5.92)	28.53 (5.87)	0.01
NK(%)	15.71 (6.62)	15.19 (6.50)	17.71 (9.45)	18.32 (9.96)	15.71 (3.62)	0.52
CD4^+^(%)	46.12 (11.46)	49.12 (11.15)	44.95 (10.96)	44.26 (10.60)	45.18 (5.61)	0.36
CD3^-^CD19^+^(%)	10.79 (5.33)	7.05 (3.25)	7.30 (4.42)	6.63 (3.30)	5.62 (1.49)	0.0002
Ig κ (mg/dl)	1,123.30 (234.57)	1,197.30 (319.33)	1,246.45 (374.01)	1,314.05 (271.20)	1,282.15 (283.25)	0.04
Ig G (g/L)	12.76 (2.93)	13.55 (4.02)	14.80 (4.24)	14.97 (3.01)	15.26 (3.15)	0.008
Ig A (g/L)	2.72 (1.08)	3.12 (0.83)	3.13 (0.85)	3.16 (0.89)	3.05 (0.83)	0.28
C4(g/L)	0.23 (0.10)	0.23 (0.09)	0.43 (0.11)	0.20 (0.06)	0.24 (0.05)	0.65
Ig λ (mg/dl)	599.95 (182.07)	634.40 (188.45)	677.15 (200.70)	713.25 (123.43)	672.10 (128.76)	0.04
Ig M (g/L)	1.38 (0.84)	1.31 (0.63)	1.36 (0.70)	1.35 (0.64)	1.48 (0.70)	0.64
*B* factor (mg/dl)	44.69 (17.87)	45.86 (10.73)	43.89 (12.19)	41.22 (9.67)	43.14 (7.74.57)	0.37
C3 (g/L)	0.98 (0.26)	0.99 (0.17)	0.97 (0.18)	0.95 (0.16)	0.97 (0.16)	0.75

Changing of biomarkers are analyzed with simple linear regression. p < 0.05 indicates slope deviation from zero.

**Figure 2:**
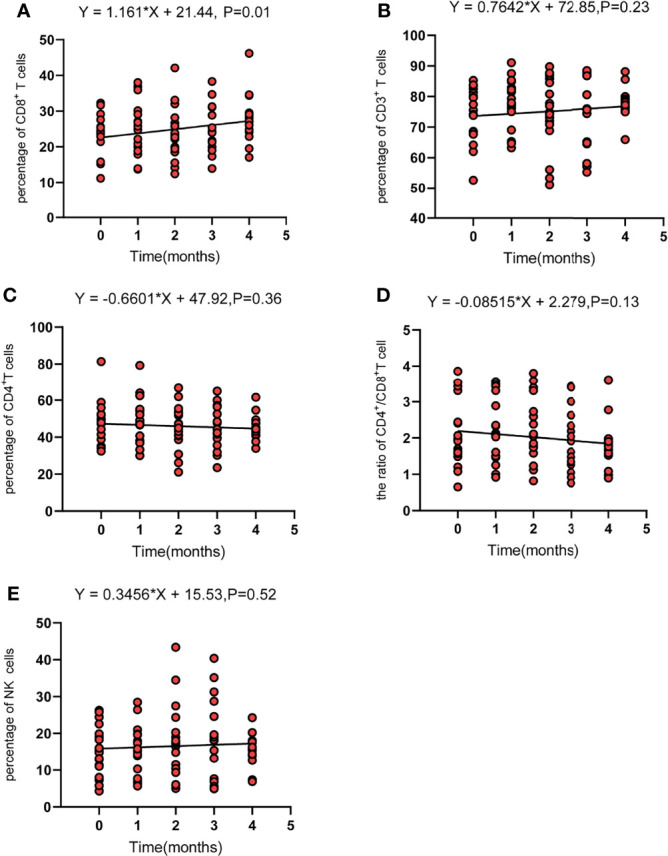
The dynamic change of CD8 T cells (CD3^+^CD8^+^) **(A)**, total T cells (CD3^+^) **(B)**, CD4 T cells (CD3^+^CD4^+^) **(C)**, the CD4^+^/CD8^+^ ratio **(D)**, and NK cells (CD3^-^CD16^+^CD56^+^) **(E)**. Fit a line with simple linear regression. p < 0.05 indicates slope deviation from zero.

**Figure 3:**
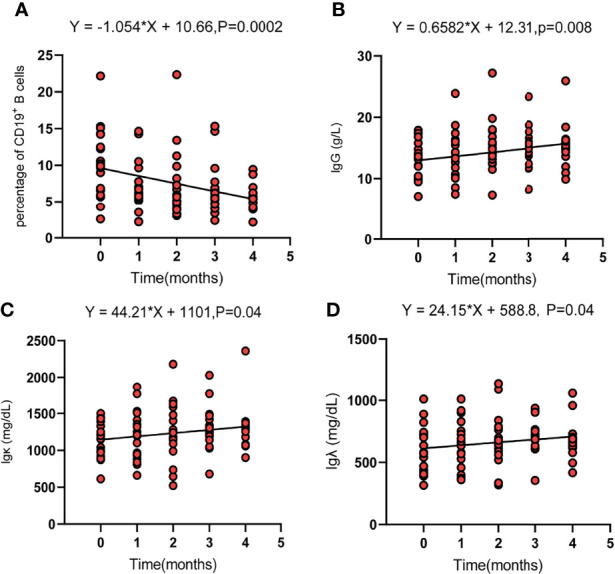
The dynamic change trend of circulating parameters of B cells (CD19^+^) **(A)**, Ig G **(B)**, Ig κ **(C)**, and Ig λ **(D)**. Fit a line with simple linear regression. p < 0.05 indicates slope deviation from zero.

**Figure 4:**
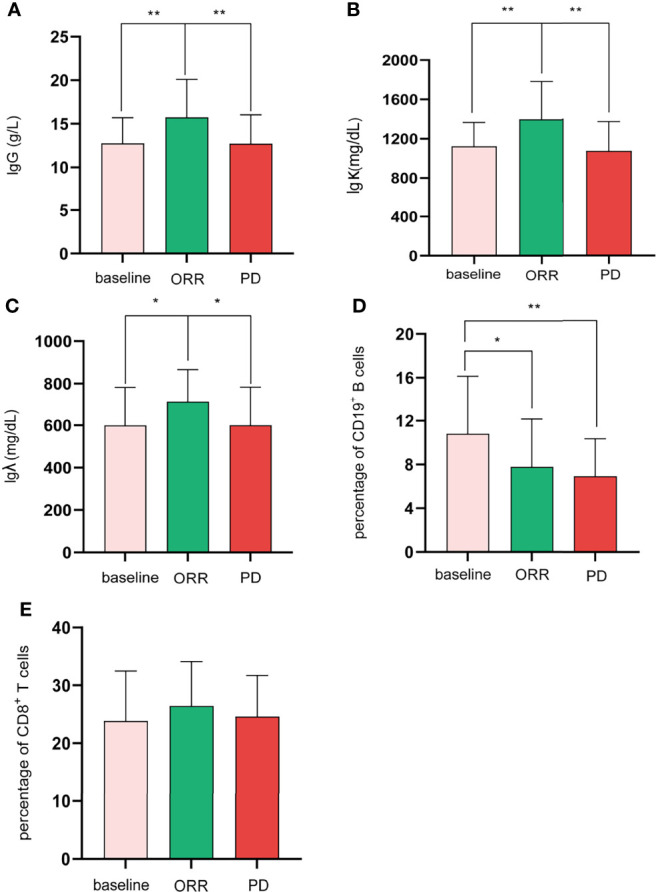
For the difference among baseline, ORR, and PD, Shapiro–Wilk tests were performed to determine the normality of the data distribution. Ig G **(A)**, Igk **(B)**, CD19^+^ B cells **(D)**, and CD8^+^ T cells **(E)**, one-way ANOVA; Ig λ **(C)**, Mann–Whitney test; *p < 0.05; **p < 0.01.

Regarding the changes in parameters during the treatment, we investigated dynamic change trends of cellular immune and humoral immune components from baseline to 4 months after treatments. In cellular immunity, CD8^+^ T cells increased significantly (*y* = 1.161X+21.44, *p* = 0.01). In addition, although the percentage of CD3^+^ T cells and NK cells showed an increasing trend, the level of CD4^+^T cells and CD4^+^/CD8^+^ ratio showed a decreasing trend, and the differences were not statistically significant (**Figure 2**). Interestingly, the percentage of B cells (CD3^-^CD19^+^) was decreased during treatment (*y* = −1.054X+10.66, *p* = 0.0002). In contrast, percentages of Ig G, Ig κ, and Ig λ increased during treatment (Ig G, *y* = 0.6582X+12.31, *p* = 0.008; Ig κ, *y* = 44.21X+1101, *p* = 0.04; Ig λ, *y* = 24.15X+588.8, *p* = 0.04) (**Figure 3**). Therefore, we analyzed CD8^+^ T cells, B cells, Ig G, Ig κ, and Ig λ at three different time points: baseline, the time of response (ORR), and the time of tumor progression (PD). As expected, we found that Ig G, Ig κ, and Ig λ increased at the time of response, and decreased again to the level of baseline with tumor progression. B cells decreased after treatment regardless of the response or tumor progression, and CD8^+^ T cells did not show relationships with the response (**Figure 4**).

## Discussion

Locoregional therapies can be combined with ICI-based immunotherapy due to their immune stimulation (24). However, combination strategies remain an undetermined challenge. In the current study, the combination of TACE+ICIs+TKIs resulted in an ORR of 52.8% and a DCR of 81.1%, with a TTP of 8.0 months and a PFS of 8.5 months. This combination therapy may have the ability to stimulate systemic immune response because circulating CD8^+^ T cells and Ig G antibodies increased significantly after treatment.

TACE has been used to treat uHCC worldwide. Repeated TACE is often necessary to maximize efficacy (1). The efficacy of TACE, however, waned over time, possibly due to the worsened hypoxia and immunosuppressive TME of residual tumor tissue following TACE (24). Antiangiogenic therapy, such as TKIs, can alleviate hypoxia and immunosuppressive TME through “tumor vascular normalization” (12). Therefore, TACE+ICIs+TKIs should be a promising combination therapy for uHCC. In this study, we found an ORR of 52.8%, which was better than the ORR of 33% in IMbrave-150 (25). The median OS in our cohort was not reached. We identified that the TTP was 8.0 months, and the PFS was 8.5 months. Zhan et al. (26) examined ICIs in combination with transarterial radioembolization (TARE) and reported that both TTP and PFS were 5. 7 months. In another study, TACE or TARE in combination with ICIs was studied in 29 patients with BCLC B or C HCC (27). The ORR of all patients was 45% in the first month, while the TTP was 4.3 months in the TACE plus ICIs cohort. Recently, Liu et al. (28) reported that a cohort of 22 patients with advanced HCC received TACE plus lenvatinib and camrelizumab. At the third month, the ORR and DCR were 72.6% and 95.5%, respectively, and the median PFS was 11.4 months. In another cohort of 62 patients treated with TACE plus lenvatinib and anti-PD-1 therapy (29), they reported an ORR of 80.6%, and 32 (53.2%) patients successfully converted to resectable HCC; among these patients, 29 patients underwent resection, and 16 patients achieved a pathological complete response. Although comparisons between different studies should be cautious, these data indicated an encouraging efficacy of this triple therapy for uHCC.

In the current study, treatment-related AEs occurred in 85% of patients. The incidence of AEs was in accordance with the other triple therapy cohort (29), which reported AEs of 74.2%. AEs of higher grade (grade ≥3) were 14.5% in their cohort and 28% in our cohort. No patient died due to AEs in either cohort. Together, this triple combination therapy appears to have an acceptable safety profile.

In HCC, previous studies have studied the change in T-cell subsets in peripheral blood after TACE. Treg cells decreased at 1 to 2 weeks after Gelatin Sponge Microparticles TACE (GSMs-TACE), which continued to decline at 3 to 5 weeks postoperatively (17). As Treg cells represent suppressor of anti-tumor immunity, GSMs-TACE could exert a positive effect on the immune function of HCC patients. However, CD8+T cells were relatively stable 1 week and 1 month after TACE (30) or RFA (31). In combined therapy, the previous study of tremelimumab combined with RFA or TACE, a significant increase in circulating CD8^+^ T cells was observed until 3 months postoperatively (16). CD8+T cells were also significantly increased in the present study. These data supported combination therapy as having the ability to activate systemic immune function. Different treatments may have different effects on the function of systemic immunity. Circulating T cells and NK cells were decreased in patients with breast cancer after chemotherapy (32). Similar results were found in a study of HCC treated with stereotactic body radiation therapy (SBRT), with reductions in CD3^+^, CD8^+^, CD4^+^ T cells and NK cells at 10 days after SBRT treatment (33). The decreased lymphocyte subsets might represent immune dysfunction. In our cohort, in addition to CD8^+^ T cells, CD3^+^ T cells and NK cells also increased during treatment. Taken together, the dynamic changes in peripheral blood T cell subsets in our cohort may reflect the activation of cellular immunity.

Patients with various cancer types receive ICI-based combination immunotherapy (19), and most of the patients exhibit a decrease in circulating B cells. In a study of patients with advanced melanoma receiving immunotherapy (anti-CTLA4, anti-PD1, or combination therapy) (34), they reported a significant decrease in circulating B cells in the combination therapy cohort, but not in patients receiving anti-CTLA4 or anti-PD1 therapy. Therefore, immune-based combination therapy could elicit a decrease in peripheral B cells, but the cause is unclear. In the current study, we found that the decrease in circulating B cells was accompanied by an increase in Ig G, Ig κ, and Ig λ. B cells migrate from the bone marrow to the secondary lymphoid organs (SLOs) and undergo isotype switching to Ig G after being activated by antigens (18, 35). Therefore, the reduction in circulating B cells may be due to the isotype switch to antibodies. indicating that humoral immunity plays an important role in this triple therapy of TACE+ICIs+TKIs.

Predictive biomarkers are still an unmet demand in HCC. In the current study, CD8+ T cells and B cells changed significantly after treatment. However, they were not associated with the response. Interestingly, we observed that Ig G, Ig λ, and Ig κ changed significantly and were associated with response. Therefore, circulating Ig G, Ig λ, and Ig κ can serve as potential biomarkers.

Our study had several limitations. First, only 20 cases were available for biomarker analysis. Perhaps due to the limited samples, there were no parameters related to survival. Second, the interval between TACE and ICIs was not fixed, which might affect the final clinical outcomes. However, the optimal interval between TACE and ICIs is still unclear now, which might be an issue to be further explored. Third, the Ig G antibody subtypes and B-cell phenotypes were not analyzed in the current study. Total B cells and Ig G lack specificity, and further research is ongoing.

In conclusion, TACE+ICIs+TKIs showed considerable efficacy in patients with uHCC. Triple therapy activated not only cell immunity but also humoral immune activation. Circulating Ig G, Ig λ, and Ig κ can serve as potential biomarkers.

## Data Availability Statement

The original contributions presented in the study are included in the article/supplementary material. Further inquiries can be directed to the corresponding authors.

## Ethics Statement

The studies involving human participants were reviewed and approved by the Institutional Review Board of the First Affiliated Hospital of Soochow University. The patients/participants provided their written informed consent to participate in this study.

## Author Contributions

X-LZ and W-SW contributed to the study concept and design. FY, G-LX, J-TH, YY, WX, W-CL, SZ, and JY contributed to the acquisition of clinical data. FY wrote the first draft of the manuscript. X-LZ, W-SW, BY-Z, and JS supervised and oversaw the study. HP-S and FY contributed to the statistical analysis. All authors contributed to the article and approved the submitted version.

## Funding

This study was supported by the Key R&D Program (Social Development) Project of Jiangsu Province (BE2021648), the National Natural Science Foundation of China (81771945), and the Heng Rui Interventional Research Fund (HRIRF-20190C047), Clinical trial registration number: ChiCTR1900027247, and the Project of Wuxi Institute of Translational Medicine (LCYJ202222).

## Conflict of Interest

The authors declare that the research was conducted in the absence of any commercial or financial relationships that could be construed as a potential conflict of interest.

## Publisher’s Note

All claims expressed in this article are solely those of the authors and do not necessarily represent those of their affiliated organizations, or those of the publisher, the editors and the reviewers. Any product that may be evaluated in this article, or claim that may be made by its manufacturer, is not guaranteed or endorsed by the publisher.

## Abbreviations

uHCC, unresectable hepatocellular carcinoma; TME, tumor microenvironment; TACE, transarterial chemoembolization; ICIs, Immune checkpoint inhibitors; TKIs, tyrosine kinase inhibitors; mRECIST, Modified Response Evaluation Criteria in Solid Tumor; ECOG-PS, Eastern Cooperative Oncology Group performance status score; BCLC, Barcelona Clinic Liver Cancer.
